# Design, Synthesis, and Characterization of an Amphiphilic Lipoic Acid-Based Ru(III) Complex as a Versatile Tool for the Functionalization of Different Nanosystems

**DOI:** 10.3390/molecules28155775

**Published:** 2023-07-31

**Authors:** Claudia Riccardi, Chiara Platella, Domenica Musumeci, Daniela Montesarchio

**Affiliations:** 1 Department of Chemical Sciences, University of Napoli Federico II, 80126 Napoli, Italy; claudia.riccardi@unina.it (C.R.); chiara.platella@unina.it (C.P.); domenica.musumeci@unina.it (D.M.); 2 Institute of Biostructure and Bioimaging (IBB), CNR, 80145 Napoli, Italy; 3 CINMPIS—Consorzio Interuniversitario Nazionale di Ricerca in Metodologie e Processi Innovativi di Sintesi, Via E. Orabona 4, 70125 Bari, Italy

**Keywords:** Ru(III) complexes, amphiphilic compounds, nucleolipids, lipoic acid, chemical synthesis, half-life time

## Abstract

Ru-based chemotherapy is emerging as an effective alternative to the well-established Pt-based one, typically associated with high toxicity. In this context, our recent efforts were devoted to the preparation of nucleolipid-based Ru(III) complexes able to form, under physiological conditions, supramolecular aggregates which can efficiently prevent metal deactivation and convey Ru(III) inside the cells where it exerts its activity. Within an interdisciplinary program for the development of multifunctional nanoparticles for theranostic applications, we here report the design, synthesis, and characterization of a novel functionalized Ru(III) salt, carrying a lipoic acid moiety in the nucleolipid-based scaffold to allow its incorporation onto metal-based nanoparticles.

## 1. Introduction

Transition metal-based drugs have aroused increasing attention in medicinal chemistry as anticancer agents for their potential value for both therapeutic and diagnostic applications [1,2,3,4]. Recently, their interest was extended to the treatment of neurodegenerative diseases as probes or inhibitors of specific biomolecules [5,6,7,8,9,10].

Among metal-containing compounds, Ru(III)-based complexes are among the most attractive molecules in anticancer strategies for their favorable anticancer or antimetastatic properties associated with good pharmacological profiles [11,12,13], as demonstrated for some iconic Ru(III) complexes, such as NAMI-A [14,15], KP1019 [16,17,18,19], and NKP-1339 [20,21,22,23], which reached advanced clinical evaluation (Supplementary Materials: Figure S1).

However, all these low molecular weight Ru(III) compounds are typically poorly stable in biological media, where hydrolysis can occur, leading to the formation of poly-oxo species as a result of the progressive substitution of the most labile chloride ligands of the metal center [24,25,26,27,28].

To retard this process, at least until the drug has been effectively internalized into cells [29], a common, extensively used approach is based on the choice of metal coordinating ligands less susceptible to the displacement operated by water molecules. In addition, ligands to be preferred should be molecules able to favor the self-assembly of the final complex in solution and to protect the metal core [30,31,32,33,34,35]. 

**Figure 1:**
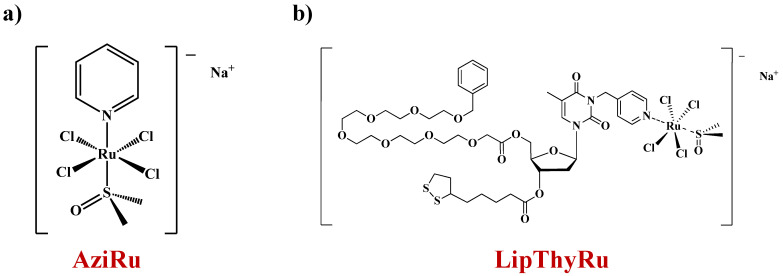
Chemical structures of (**a**) the Ru(III) complex AziRu and (**b**) the amphiphilic nucleolipid-based Ru(III) complex LipThyRu, as indicated.

In this context, starting from a structurally-related NAMI-A analog, called AziRu (Figure 1a) [36,37], we previously designed and synthesized a library of highly functionalized nucleolipid- [29,38] or aminoacyl lipid-based [39] Ru(III)-containing complexes. The resulting derivatives have been then co-formulated with biocompatible vesicle-forming lipids, such as POPC or DOTAP, obtaining a mini-library of various Ru(III)-loaded liposomes [29,36,40,41] characterized by high antiproliferative activity on human cancer cells, especially of breast cancer origin, and no relevant toxicity on control healthy cell lines [29,42]. These liposomes also proved to be effective and selective in vivo in preclinical evaluation studies in suitable breast cancer xenografts mouse models [43,44].

In parallel, one of the most promising nucleolipid-based Ru(III) complexes, named HoThyRu (Figure S1), was also embedded in niosomes further functionalized with the nucleolin-targeting AS1411 aptamer, allowing selective recognition of cancer cells. Tested on both cancer and healthy cell lines, niosomes decorated with both molecules showed promising antiproliferative activity on human cervix adenocarcinoma cells (HeLa), where AS1411 was proven to markedly enhance the bioactivity of the Ru(III)-containing niosomes [45].

Stimulated by the excellent in vitro and in vivo properties of the proposed formulations and aiming at expanding the repertoire and chemical diversity of the available Ru(III)-containing nanosystems [42], we herein propose a novel nucleolipid-based Ru(III) complex carrying a lipoic acid moiety, suitable for the functionalization of different nanostructured materials. This compound was named LipThyRu, following the acronym taken from the main structural motifs present in its molecular skeleton: Lipoic acid–Thymidine–Ruthenium salt (Figure 1b).

## 2. Results and Discussion

### 2.1. Design of LipThyRu

With a general structure similar to the previously described nucleolipid-based compounds [29,38], LipThyRu is a thymidine derivative carrying a pyridine methyl arm at the N-3 position of the nucleobase, inserted as the anchoring ligand for the Ru(III) complex. This nucleoside has been further derivatized with a hydrophilic heptaethylene glycol chain at the 5′ position and a lipophilic chain, i.e., a lipoic acid residue, at the 3′ position. As for the previously developed systems [29,38,39], the use of a hydrophilic moiety, such as an oligoethyleneglycol group, is intended not only to optimize the “hydrophilic/lipophilic balance” within the hybrid molecule but also—at a functional level—to prevent extracellular clearance by the reticuloendothelial system [46,47]. On the other hand, the lipophilic component of the nucleolipid-based Ru(III) complex is necessary in our design to ensure its self-assembly in aqueous solution but also to establish specific interactions (e.g., hydrophobic, electrostatic) with suitable nanoplatforms, which can, thus, enable it to give stable formulations.

In this frame, the presence of the lipoic acid disulfide bond in LipThyRu can be exploited to allow its easy immobilization onto gold- [48,49] or silver-based [50,51] nanoparticles, thanks to the high affinity of sulfur for these metals, forming very stable covalent bonds, but also metal oxide nanoparticles (NPs), such as zinc oxide ones [52]. In addition, exploiting the ring opening disulfide-exchange polymerization (RODEP) process [53], LipThyRu could also be used for the spontaneous formation of self-organized polymers, in analogy to a recently reported study [54], considering that the concentration of free thiols is relatively low in extracellular fluids, but reaches significant values in the cytoplasm (1–10 mM) [55].

Thus, LipThyRu—carrying useful decorations which in principle allow its easy incorporation onto different nanoplatforms—is here proposed as a versatile tool in the construction of multifunctional nanosystems.

The decoration with the lipophilic and hydrophilic chains on the sugar hydroxyl moieties was performed through simple ester bonds, which are realized in high yields by classical DCC/DMAP activation of the selected carboxylic acids [56,57]. These chemical linkages are typically stable in neutral solutions and extracellular media but are rapidly cleaved within the cell by cellular esterases, releasing the active portion of the molecule, i.e., the AziRu-like compound.

### 2.2. Synthesis and Characterization of LipThyRu

**Scheme 1:**
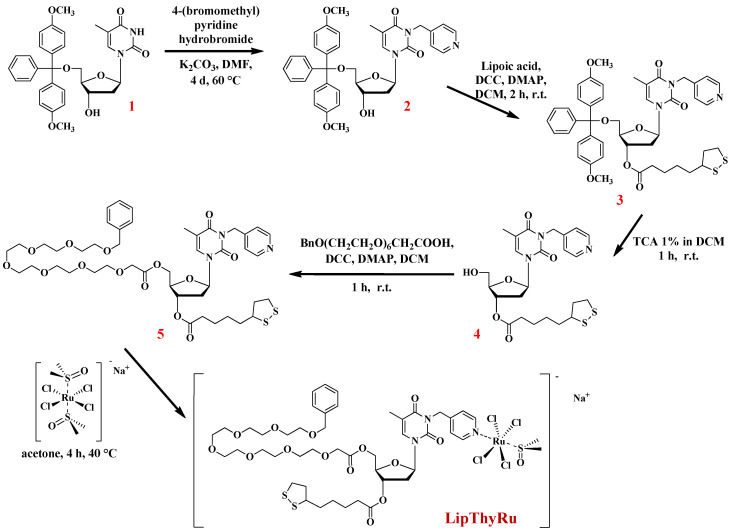
Synthetic procedure for the preparation of the nucleolipid-based Ru(III) complex LipThyRu. DMF = N,N-dimethylformamide; DCC = N,N′-dicyclohexylcarbodiimide; DMAP = 4-(dimethylamino)pyridine; TCA = trichloroacetic acid; Bn = benzyl group.

The synthesis of the target LipThyRu was realized by a straightforward and high yielding strategy based on well-established chemistry, involving the use of easy-to-handle, commercially available reagents (Scheme 1).

The insertion of the pyridine moiety on the thymidine scaffold was achieved by treatment of the commercially available 5′-O-(4,4′-dimethoxytriphenylmethyl)thymidine **1** with a slight excess of 4-(bromomethyl)pyridine in DMF in the presence of K_2_CO_3_ as base. In this reaction, the imide N-3 of thymidine has a relatively acidic proton (pK_a_ ca. 8) and could be easily deprotonated, thus undergoing a nucleophilic substitution and giving alkylated compound **2** in 59% isolated yields. Under the used conditions, the less reactive 3′-hydroxyl of the sugar moiety did not compete in the alkylation reaction, remaining available for the next coupling step. Indeed, this group was then coupled with lipoic acid using DCC/DMAP as condensing agents [56,57], affording compound **3** in 77% isolated yields after chromatographic purification on a silica gel column. The successive dimethoxytrityl (DMT) removal, realized under acidic conditions (1% TCA in CH_2_Cl_2_), gave compound **4** in almost quantitative yields (99%). Next, the condensation with the heptaethylene glycol derivative BnO(CH_2_CH_2_O)_6_CH_2_COOH—synthesized using previously described protocols [29,38,45]—by means of DCC/DMAP activation allowed obtaining the target nucleolipid **5**. The last step was a ligand exchange reaction between the pyridine moiety of nucleolipid **5** and the dimethylsulfoxide ligand of the Na^+^ [*trans*-RuCl_4_(DMSO)_2_]^−^ salt, prepared according to reported procedures [58,59]. The reaction was performed at 40 °C using acetone as solvent, affording the desired LipThyRu in 71% yields. Following this synthetic procedure, LipThyRu was obtained in 24% overall yields for 5 steps.

All the synthetic intermediates and the final nucleolipid have been characterized by ^1^H and ^13^C NMR spectroscopy, as well as by ESI-MS spectrometry, which in all cases confirmed the identity and purity of the target compounds. Signals observed in the ^1^H NMR spectrum of LipThyRu (Figure S2a) are diagnostic of the effective complex formation [36,39,60]. Indeed the presence of the paramagnetic ruthenium(III) ion produces a remarkable upfield shift and a broadening of the protonic signals of pyridine and dimethyl sulfoxide ligands, found at *δ* = −1.8 and *δ* = −12.7 ppm, respectively, in analogy with those reported for AziRu [37,61].

In the ESI-MS spectrum, recorded in the positive ion mode, the molecular ion shows the expected mass/charge ratio and the isotopic mass distribution (Figure S2b).

### 2.3. UV–Vis Studies of LipThyRu

**Figure 2:**
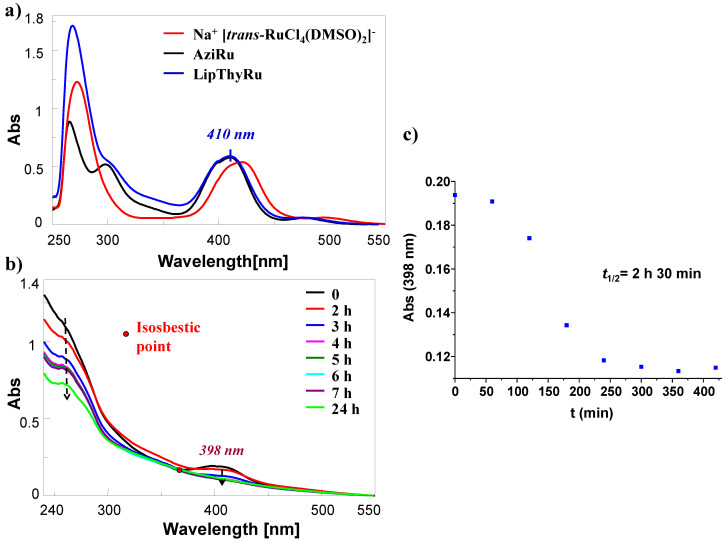
(**a**) Overlapped UV–Vis absorption spectra of freshly dissolved Na^+^ [*trans*-RuCl_4_(DMSO)_2_]^−^, AziRu, and LipThyRu in DMSO at 100 μM concentration; (**b**) overlapped UV–Vis absorption spectra of LipThyRu at 40 μM concentration in the selected saline phosphate buffer solution (10 mM phosphate buffer/100 mM NaCl, pH = 7.3), recorded at different times after dissolution (0→24 h). The red circle indicates the isosbestic point; (**c**) A_398_ values of LipThyRu at 40 μM concentration as a function of time in the selected saline phosphate buffer.

To have a complete picture of the LipThyRu features, its spectroscopic behaviour was studied. First, the UV–Vis spectrum of LipThyRu was recorded immediately after its dissolution in DMSO at 100 μM final concentration, and compared with those of AziRu [37] and its precursor Na^+^ [*trans*-RuCl_4_(DMSO)_2_]^−^ salt (Figure 2a).

The UV–Vis spectrum of LipThyRu showed the characteristic band centered at 410 nm, relative to the ligand-to-metal charge transfer transition (LMCT) for the Ru–Cl bond, in addition to overlapped bands in the 250–350 nm region, which can be attributed to the metal, the pyridine portion and the thymine base contributions. Comparing the UV–Vis spectra of LipThyRu and AziRu, the presence of the same band at ca. 400 nm confirmed the same ligands arrangement around the Ru(III) nucleus. Interestingly, this band is slightly blue-shifted with respect to the Na^+^ [*trans*-RuCl_4_(DMSO)_2_]^−^ salt—a precursor of both AziRu and LipThyRu—which carries DMSO as an apical ligand in lieu of pyridine.

These spectral features further confirmed the effective replacement of a S-Ru with a N-Ru bond, as already found for previously developed Ru(III)-containing complexes [29,39,61].

Then, the hydrolysis of LipThyRu at 50 μM concentration was monitored by UV–Vis analysis over time in a saline phosphate-buffered solution (10 mM phosphate buffer/100 mM NaCl pH = 7.3), mimicking the extracellular media. In this saline condition, the absorption spectrum of LipThyRu (Figure 2b) showed the LMCT band centered at 398 nm, slightly blue-shifted with respect to the maximum observed for the same compound after dissolution in DMSO, according to previous findings for AziRu [37,39] and other AziRu-based compounds [61]. This band gradually diminished over time, disappearing at ca. 7 h with a concomitant decrease in the absorption bands in the region between 250–300 nm (Figure 2b).

Since the first monitoring hours, an overall raising of the spectrum baseline was observed, associated with scattering phenomena, probably due to the formation of large aggregates, presumably poly-oxo species, which reached stability after 4 h (Figure 2b).

In analogy with AziRu [37], the presence of an isosbestic point is evident for LipThyRu, indicating its conversion within 3.5 h into another species, i.e., the monoaquo complex.

Reporting the A_398_ as a function of time (Figure 2c), a half-life time (*t*_1/2_) of 2.5 h is obtained, almost 3.3 times higher than that observed for AziRu (*t*_1/2_ for AziRu = 45 min [37,61]).

Interestingly, the determined *t*_1/2_ value of LipThyRu was also higher than those calculated for the previously studied nucleolipid- and aminoacyl lipid-based Ru(III) complexes [39] and is in the same range of those obtained for recently investigated lipid conjugated Ru(III)-containing compounds [61]. These outcomes suggest that the groups decorating the Ru(III) complex are effectively able to retard the formation of aquo species, resulting in a slower hydrolysis rate of LipThyRu compared to the naked AziRu.

## 3. Experimental Section

### 3.1. Materials and General Methods

All the reagents and solvents were of the highest commercially available quality and were used as received; where anhydrous conditions were required, co-evaporations with dry CH_3_CN were performed before use. All the esterification reactions were carried out in the presence of activated molecular sieves and under an argon atmosphere. TLC analyses were carried out on silica gel plates from Macherey-Nagel (60, F254). Reaction products on TLC plates were visualized by UV-light and then by treatment with an oxidant acidic solution (acetic acid/water/sulfuric acid, 10:4:5, *v*/*v*). For the column chromatography purifications, silica gel from Macherey-Nagel (Kieselgel 60, 0.063–0.200 mm) was used.

The ruthenium complexes Na^+^ [*trans*-RuCl_4_(DMSO)_2_]^−^ [58,59] and AziRu [37,62] were synthesized according to described protocols starting from the commercially available ruthenium trichloride (Sigma Aldrich). Their identity and purity were confirmed by NMR and IR spectroscopic analyses as well as by melting point measurements, which in all cases were in accordance with the literature data. Also, the heptaethylene glycol derivative BnO(CH_2_CH_2_O)_6_CH_2_COOH was prepared using previously reported protocols [29,38,45]. For this compound, the obtained spectroscopic (^1^H- and ^13^C-NMR) and spectrometric (ESI-MS) data were in perfect agreement with those reported in the literature [38].

NMR spectra were recorded on a Bruker WM-400. All the *chemical shifts* (δ) are expressed in ppm with respect to the residual solvent signal for both ^1^H NMR [CDCl_3_ = 7.26 ppm, (CD_3_)_2_CO = 2.05 ppm,] and ^13^C NMR (CDCl_3_ = 76.9 ppm). All the coupling constants (*J*) are quoted in Hertz (Hz). Peaks assignments were carried out based on standard ^1^H-^1^H COSY and HSQC experiments. The following abbreviations were used to explain the multiplicities: s = singlet; d = doublet; t = triplet; q = quartet; m = multiplet; b = broad.

For the ESI-MS analyses, a Waters Micromass ZQ instrument—equipped with an electrospray source—was used in the positive and/or negative mode.

UV–Vis measurements were performed on a JASCO V-530 UV–Vis spectrophotometer equipped with Peltier Thermostat JASCO ETC-505T, by using 1 cm path length cuvette. The spectra were recorded at r.t. in the range 200–600 nm with a medium response, a scanning speed of 100 nm/min, and a 2.0 nm bandwidth with the appropriate baseline subtracted. All the UV–Vis spectra were averaged over 3 scans, and each experiment was performed in triplicate. The target compounds were analyzed from freshly dissolved samples at 100 μM concentration in DMSO or at 50 μM concentration in the selected saline-buffered solution (10 mM KH_2_PO_4_/K_2_HPO_4_, 100 mM NaCl, pH = 7.3). Samples prepared in buffer solutions were also monitored over time up to 24 h to determine the stability of LipThyRu to the hydrolysis process. The *t*_1/2_ value was determined as the time at which the absorbance value at λ ca. 400 nm reached 50% of its initial value (i.e., at t = 0), measured immediately after dissolution of the sample in the proper buffer [39,61].

### 3.2. Synthesis and Characterization of LipThyRu

#### 3.2.1. Synthesis of 3-(4-pyridylmethyl)-5′-O-(4,4′-dimethoxytriphenylmethyl)-thymidine (2)

5′-O-(4,4′-dimethoxytriphenylmethyl)thymidine **1** (121 mg, 0.22 mmol) was dissolved in 8 mL of dry DMF. K_2_CO_3_ (92.0 mg, 0.67 mmol) and 4-(bromomethyl)pyridine hydrobromide (84.2 mg, 0.33 mmol) were then added to the reaction mixture and left at 60 °C under stirring. After 4 d, TLC analysis indicated the presence of the desired product, and the reaction was quenched by removing the solvent under reduced pressure. First, an extraction with CH_2_Cl_2_/H_2_O was performed to remove the excess of reagents. Then, the crude product was purified by chromatography on a silica gel column using AcOEt/MeOH (97:3, *v*/*v*, containing 2% of TEA), as eluent, giving desired compound **2** in 59% yield (82.5 mg, 0.13 mmol).

**2**: Oil. *R_f_* = 0.7 (AcOEt/MeOH, 95:5, *v*/*v*).

**^1^H NMR** (400 MHz, CDCl_3_, Figure S3): *δ* 8.44 (d, *J* = 5.2, 2H, 2x ***H***α Py); 7.58 (s, 1H, ***H***-6); 7.36 (d, *J* = 7.4, 2H, 2x Hβ Py); 7.32–7.15 (overlapped signals, 9H, aromatic protons of DMT); 6.74 (d, *J* = 8.7, 4H, 4 aromatic protons of DMT), 6.33 (t, *J* = 7.4 and 6.7, 1H, ***H***-1′); 5.05 (s, 2H, -C***H_2_***Py); 4.52 (m, 1H, ***H***-3′); 4.0 (m, 1H, ***H***-4′); 3.71 [s, 6H, 2x (-OC***H_3_***) of DMT group]; 3.41–3.28 (m, 2H, ***H_2_***-5′); 2.55-2.23 (m, 2H, ***H***-2′); 1.39 (s, 3H, C***H_3_***-Thy).

#### 3.2.2. Synthesis of 3-(4-pyridylmethyl)-3′-O-lipoyl-5′-O-(4,4′-dimethoxytriphenylmethyl)-thymidine (3)

Alkylated compound **2** (70.0 mg, 0.11 mmol) was dissolved in 2 mL of dry CH_2_Cl_2_ and then DMAP (40.0 mg, 0.33 mmol), lipoic acid (34.0 mg, 0.16 mmol), and DCC (68.0 mg, 0.33 mmol) were sequentially added. After stirring for 2 h at r.t., TLC analysis indicated the complete disappearance of the starting materials. Thus, the reaction mixture was concentrated under reduced pressure; the crude product was purified by chromatography on a silica gel column using CHCl_3_/MeOH (95:5, *v*/*v*, containing 2% of TEA) as eluent, providing desired compound **3** in 77% yield (66.0 mg, 0.08 mmol).

**3**: Oil. *R_f_* = 0.8 (AcOEt/MeOH, 95:5, *v*/*v*).

**^1^H NMR** (400 MHz, CDCl_3_, Figure S4): *δ* 8.55 (d, *J* = 5.2, 2H, 2x ***H***α Py); 7.67 (s, 1H, ***H***-6); 7.39–7.28 (overlapped signals, 11H, 2x ***H***β Py and 9 aromatic protons of DMT); 6.85 (d, *J* = 8.7, 4H, 4 aromatic protons of DMT); 6.45 (dd, *J* = 7.4 and 6.7, 1H, ***H***-1′); 5.45 (m, 1H, ***H***-3′); 5.12 (s, 2H, -C***H_2_***Py); 4.11 (m, 1H, ***H***-4′); 3.80 [s, 6H, 2x (-OC***H*_3_**) of DMT]; 3.58 (m, 1H, C***H***-S); 3.49 (m, 2H, ***H_2_***-5′); 3.23–3.11 (m, 2H, C***H_2_***-S); 2.50–2.46 (m, 3H, ***H_2_***-2′ and C***H_2_***a-CH_2_-S); 2.35 (t, *J* = 7.4, 2H, -C***H*_2_**-C=O lipoic residue); 1.95–1.90 (m, 1H, C***H_2_***b-CH_2_-S); 1.73–1.46 (m, 6H, 3x C***H_2_*** lipoic residue); 1.42 (s, 3H, C***H_3_***-Thy).

**^13^C NMR** (100 MHz, CDCl_3_, Figure S5): *δ* 172.6 (***C***=O lipoic ester); 163.0 (***C***-4 Thy); 158.6, 133.8, 129.9, 127.9, 127.8, 127.9 and 113.1 (aromatic carbons of DMT); 150.7 (***C***-2 Thy); 149.7 (2x ***C***α Py); 145.2 (***C***γ Py); 135.0 (***C***-6 Thy); 123.3 (2x ***C***β Py); 110.6 (***C***-5 Thy); 87.0 (quaternary carbon of DMT); 85.0 (***C***-1′); 83.9 (***C***-4′); 75.0 (***C***-3′); 63.4 (***C***-5′); 56.0 (***C***H-S); 55.1 [2x (O***C***H_3_) of DMT]; 43.4 (-***C***H_2_Py); 40.0 (***C***H_2_-S); 38.3 (***C***H_2_-C=O lipoic residue); 37.9 (***C***-2′); 34.7, 28.4, 25.2, 24.7 (overlapped signals, 4 aliphatic carbons of lipoic residue); 12.1 (***C***H_3_-Thy).

**ESI-MS** (positive ions, Figure S6): calculated for C_45_H_49_N_3_O_8_S_2_, 823.3; found *m*/*z*: 862.49 [M+K^+^].

#### 3.2.3. Synthesis of 3-(4-pyridylmethyl)-3′-O-lipoyl-thymidine (4)

Compound **3** (237 mg, 0.29 mmol) was dissolved in 2 mL of a 1% TCA solution in CH_2_Cl_2_. Upon addition of the acid, the reaction mixture showed an intense yellow-orange colour, typical of the dimethoxytrityl cation, and was left under stirring at r.t. After 1 h, TLC monitoring indicated the complete disappearance of the starting compound. Thus, the reaction was quenched by adding a few drops of MeOH until complete decoloration, and finally TEA was added to neutralize the solution. Then the reaction mixture was concentrated in vacuo and purified by chromatography on a silica gel column eluted with CHCl_3_/MeOH (99:1, *v*/*v*), giving the desired compound **4** in almost quantitative yields (155 mg, 0.29 mmol).

**4**: Yellow oil. *R_f_ =* 0.4 (CHCl_3_/MeOH, 95:5, *v*/*v*).

**^1^H NMR** (400 MHz, CDCl_3_, Figure S7): *δ* 8.53 (d, *J* = 5.2, 2H, 2x ***H***α Py); 7.73 (s, 1H, ***H***-6); 7.32 (d, 2H, 2x ***H***β Py); 6.34 (dd, *J* = 7.4 and 6.7, 1H, ***H***-1′); 5.37 (m, 1H, ***H***-3′); 5.12 (s, 2H, -C***H_2_***Py); 4.10 (m, 1H, ***H***-4′); 3.93 (m, 2H, ***H_2_***-5′); 3.59 (m, 1H, C***H***-S); 3.23–3.13 (m, 2H, C***H_2_***-S); 2.50 (m, 1H, C***H_2_***a-CH_2_-S); 2.38–2.35 (m, 4H, ***H_2_***-2′, C***H_2_***-C=O lipoic residue); 1.95 (m, 1H, C***H_2_***b-CH_2_-S); 1.75–1.65 (m, 4H, 2x C***H_2_*** lipoic residue); 1.51–1.41 (m, 2H, C***H_2_***-CH-S); 1.33(s, 3H, C***H_3_***-Thy).

**^13^C NMR** (100 MHz, CDCl_3_, Figure S8): *δ* 173.1 (***C***=O lipoic ester); 166.0 (***C***-4 Thy); 150.3 (2x ***C***α Py); 145.9 (***C***-2 Thy); 134.9 (***C***γ Py); 123.5 (2x ***C***β Py); 113.7 (***C***-6 Thy); 109.4 (***C***-5 Thy); 86.5 (***C***-1′); 85.2 (***C***-4′); 73.7 (***C***-3′); 62.5 (***C***-5′); 56.4(***C***H-S); 43.6 (-***C***H_2_Py); 40.3 (***C***H_2_-S); 38.6 (***C***H_2_-C=O lipoic residue); 37.7 (***C***-2′); 34.6–24.5 (overlapped signals, 4 aliphatic carbons of lipoic residue); 12.2 (***C***H_3_-Thy).

**ESI-MS** (positive ions, Figure S9): calculated for C_24_H_31_N_3_O_6_S_2_, 521.17; found *m*/*z*: 544.34 [M+Na^+^]; 560.38 [M+K^+^].

#### 3.2.4. Synthesis of 3-(4-pyridylmethyl)-3′-O-lipoyl-5′-O-(benzyloxy)hexaethylene glycol acetyl-thymidine (5)

To compound **4** (35.0 mg, 0.07 mmol), dissolved in 1 mL of dry CH_2_Cl_2_, DMAP (16.4 mg, 0.13 mmol), derivative **8**, i.e., BnO(CH_2_CH_2_O)_6_CH_2_COOH (43.0 mg, 0.10 mmol), and DCC (28.0 mg, 0.14 mmol) were sequentially added, and the resulting reaction mixture was left under stirring at r.t.. After 1 h, TLC analysis indicated the formation of a new product, so the solvent was removed under reduced pressure and the crude product was purified by chromatography on a silica gel column eluted with CHCl_3_/MeOH (95:5, *v*/*v*), giving the desired compound **5** in 74% yield (48.4 mg, 0.05 mmol).

**5**: Oil. *R_f_ =* 0.8 (CHCl_3_/MeOH, 9:1, *v*/*v*).

**^1^H NMR** (400 MHz, CDCl_3_, Figure S10): *δ* 8.51 (d, *J* = 5.2, 2H, 2x ***H***α Py); 7.34–7.26 (overlapped signals, 8H, ***H***-6, 2x ***H***β Py and 5 aromatic protons of Bn); 6.34 (dd, *J* = 7.4 and 6.7, 1H, ***H***-1′); 5.20 (m, 1H, ***H***-3′); 5.08 (s, 2H, -C***H_2_***Py); 4.54 (s, 2H, -C***H_2_***Ph); 4.42 (m, 2H, ***H_2_***-5′); 4.26–4.12 (overlapped signals, 3H, ***H***-4′ and -OC***H_2_***COO-C5′); 3.78–3.53 [overlapped signals, 25H, 6x (-O-C***H_2_***-C***H_2_***-O-) and C***H***-S]; 3.18–3.08 (m, 2H, C***H_2_***-S); 2.37–2.32 (overlapped signals, 5H, ***H_2_***-2′, C***H_2_***-C=O lipoic residue and C***H_2_***a-CH_2_-S); 1.92 (s, 3H, ***CH_3_***-Thy); 1.90 (m, 1H, C***H_2_***b-CH_2_-S); 1.69–1.60 (m, 4H, 2x C***H_2_*** lipoic residue); 1.45 (m, 2H, C***H_2_***-CH-S lipoic residue).

**^13^C NMR** (100 MHz, CDCl_3_, Figure S11): *δ* 171.8 (***C***=O lipoic ester); 168.7 (***C***=O HEG ester); 161.9 (***C***-4 Thy); 149.7 (***C***-2 Thy); 148.9 (2x ***C***α Py); 144.3 (***C***γ Py); 137.2 (quaternary carbon of Bn); 132.4 (***C***-6 Thy); 127.2, 126.5 (aromatic carbons of Bn); 122.3 (2x ***C***β Py); 109.7 (***C***-5 Thy); 84.4 (***C***-1′); 81.0 (***C***-4′); 73.2 (***C***-3′); 72.1 (-***C***H_2_Ph); 70.0, 69.4 (O-***C***H_2_-***C***H_2_-OHEG); 68.3 (-O***C***H_2_COO-C5′); 63.1 (***C***-5′); 55.1(***C***H-S); 50.7 (***C***H_2_-S); 42.5 (-***C***H_2_Py); 37.4 (***C***H_2_-C=O lipoic residue); 35.9 (***C***-2′); 33.4–23.4 (overlapped signals, 4 aliphatic carbons of lipoic residue); 12.2 (***C***H_3_-Thy).

**ESI-MS** (positive ions, Figure S12): calculated for C_45_H_63_N_3_O_14_S_2_, 933.38; found *m*/*z*: 934.68 [M+H^+^]; 972.38 [M+K^+^].

#### 3.2.5. Synthesis of LipThyRu

Nucleolipid **5** (46.0 mg, 0.05 mmol) was dissolved in 2.5 mL of acetone and then Na^+^ [*trans*-RuCl_4_(DMSO)_2_]^−^ salt (21.0 mg, 0.05 mmol) was added under stirring at 40 °C. After 4 h the reaction, monitored via TLC, indicated the complete disappearance of both starting products and the concomitant formation of a more polar product. After removing the solvent under reduced pressure, the obtained solid was exhaustively washed with AcOEt, and able to solubilize only the target complex LipThyRu, which was, thus, recovered in 71% isolated yields as a pure compound (45.4 mg, 0.03 mmol).

**LipThyRu**: Yellow oil. *R_f_ =* 0.3 (CHCl_3_/MeOH, 9:1 *v*/*v*).

**^1^H NMR** [400 MHz, (CD_3_)_2_CO]: significant signals at *δ* -1.75 (very broad signal, Py protons); -12.7 (very broad signal, C***H_3_*** of DMSO).

**ESI-MS** (positive ions): calculated for C_47_H_69_Cl_4_N_3_NaO_15_RuS_3_^+^, 1276.16; found *m*/*z*: 1257.25 [(M-Na^+^)+2H^+^].

## 4. Conclusions

Considering the great potential of nucleolipids as a versatile class of self-assembling amphiphilic molecules [63,64,65,66,67] able to act as molecular carriers for Ru(III)-based drugs and aiming at enriching the arsenal of efficient anticancer Ru(III)-containing nanosystems, we have designed, synthesized, and characterized a novel nucleolipid-based Ru(III) complex functionalized with a residue of lipoic acid that we named LipThyRu. This appendage is intended to allow its subsequent immobilization onto metal oxide-based nanoplatforms, thanks to the high affinity of sulfur to metals and following the formation of covalent bonds. In this frame, magnetic NPs could be those of choice, being extensively used as diagnostic tools in magnetic resonance imaging (MRI) [68,69]. As an alternative, gold NPs or zinc oxide NPs can be selected for their great potential in fluorescence imaging [52]. In this way, the final Ru(III) complex-loaded nanosystem can be useful not only for therapeutic applications but also for diagnostic or theranostic purposes.

LipThyRu was fully characterized using spectroscopic and spectrometric techniques and further studied by UV spectrometry to analyze its behaviour toward the hydrolysis process, typical of Ru(III) complexes. A half-life time value almost 3.3 times higher than that observed for the Ru(III) compound AziRu, as well as higher than the previously studied nucleolipid- and aminoacyl lipid-based Ru(III) complexes [39], was found, suggesting the inserted decoration as a useful tool for both retarding the hydrolysis process and potentially allowing its binding on different metal-based nanoplatforms.

## Data Availability

Samples of the described compounds are available upon request.

## Supplementary Materials

The following supporting information can be downloaded at: https://www.mdpi.com/article/10.3390/molecules28155775/s1, Figure S1. Chemical structures of previously reported Ru(III) complexes; Figure S2. NMR and ESI-MS spectra of final LipThyRu Ru(III) complex; Figure S3. ^1^H-NMR spectrum of compound **2**; Figure S4–S6. ^1^H-NMR, ^13^C-NMR and ESI-MS spectra of compound **3**; Figure S7–S9. ^1^H-NMR, ^13^C-NMR and ESI-MS spectra of compound **4**; Figure S10–S12. ^1^H-NMR, ^13^C-NMR and ESI-MS spectra of compound **5**.

## Sample Availability

Not available.
