# Supplementary material for: Design, Synthesis, and Characterization of an Amphiphilic Lipoic Acid-Based Ru(III) Complex as a Versatile Tool for the Functionalization of Different Nanosystems
Source: Molecules. 2023 Jul 31;28(15):5775. doi: 10.3390/molecules28155775 (PMC10420320; doi:10.3390/molecules28155775)
Supplement: Supplementary file 1 [file molecules-28-05775-s001.zip › molecules-2525653-supplementary.pdf]

Supporting Information

to the manuscript:

Design, synthesis, and characterization of an amphiphilic lipoic acid-based Ru(III) complex as a versatile tool for the functionalization of different nanosystems

Claudia Riccardi [1], Chiara Platella [1], Domenica Musumeci [1,2] and Daniela Montesarchio [1,3,*]

[1] Department of Chemical Sciences, University of Napoli Federico II, 80126 Napoli, Italy; claudia.riccardi@unina.it (C.R.); chiara.platella@unina.it (C.P.); domenica.musumeci@unina.it (D.M.)
[2] Institute of Biostructure and Bioimaging (IBB), CNR, 80145 Napoli, Italy
[3] CINMPIS—Consorzio Interuniversitario Nazionale di Ricerca in Metodologie e Processi Innovativi di Sintesi, Via E. Orabona 4, 70125 Bari, Italy
* Correspondence: daniela.montesarchio@unina.it

Table of contents

Figure S1. Chemical structures of previously reported Ru(III) complexes.

Figure S2. NMR and ESI-MS spectra of final LipThyRu Ru(III) complex.

Figure S3. ^{1}H-NMR spectrum of compound **2**.

Figure S4-S6. ^{1}H-NMR, ^{13}C-NMR and ESI-MS spectra of compound **3**.

Figure S7-S9. ^{1}H-NMR, ^{13}C-NMR and ESI-MS spectra of compound **4**.

Figure S10-S12. ^{1}H-NMR, ^{13}C-NMR and ESI-MS spectra of compound **5**.

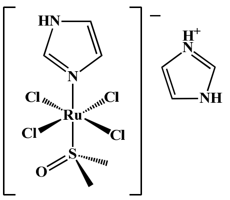

NAMI-A

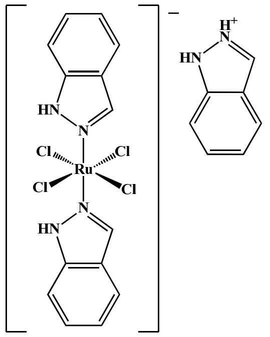

KP1019

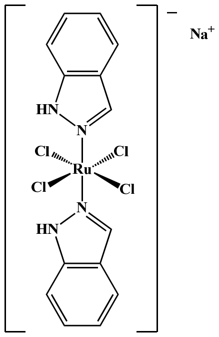

NKP1339

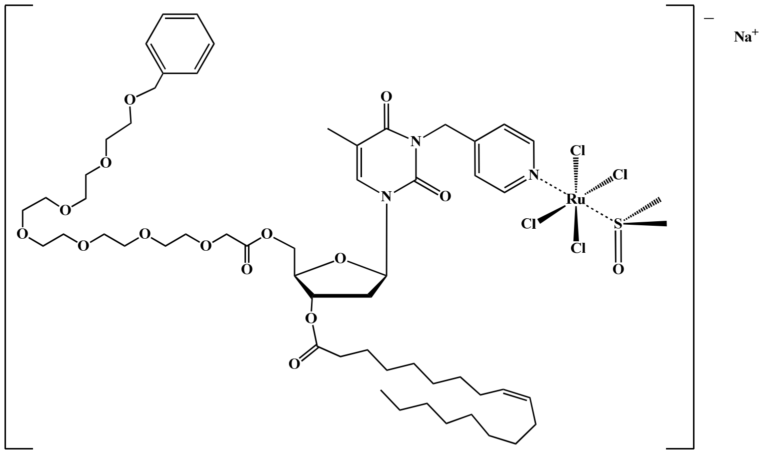

HoThyRu

Figure S1. Chemical structures of NAMI-A, KP1019, NKP-1339 and HoThyRu, as indicated.

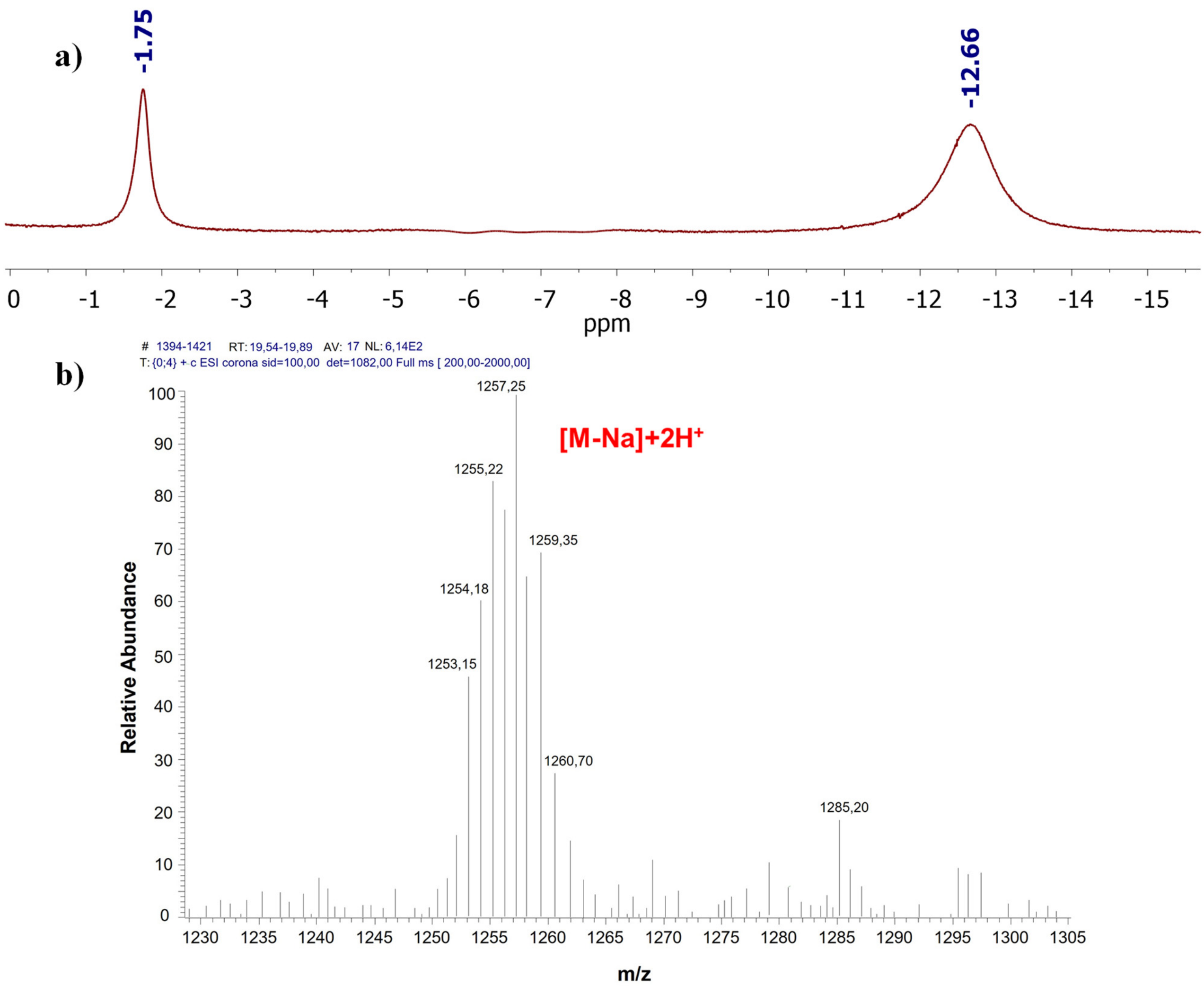

Figure S2. a) Expansion of the upfield region of the ^{1}H NMR spectrum of the nucleolipid-based Ru(III) complex LipThyRu [400 MHz, $(CD_3)_2CO$]; **b**) ESI-MS spectrum (positive ions) of LipThyRu.

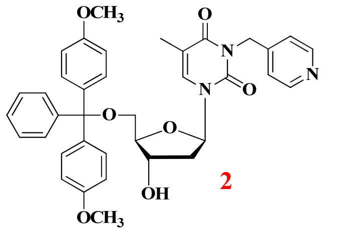

2

3-(4-pyridylmethyl)-5′-O-(4,4′-dimethoxytriphenylmethyl)-thymidine

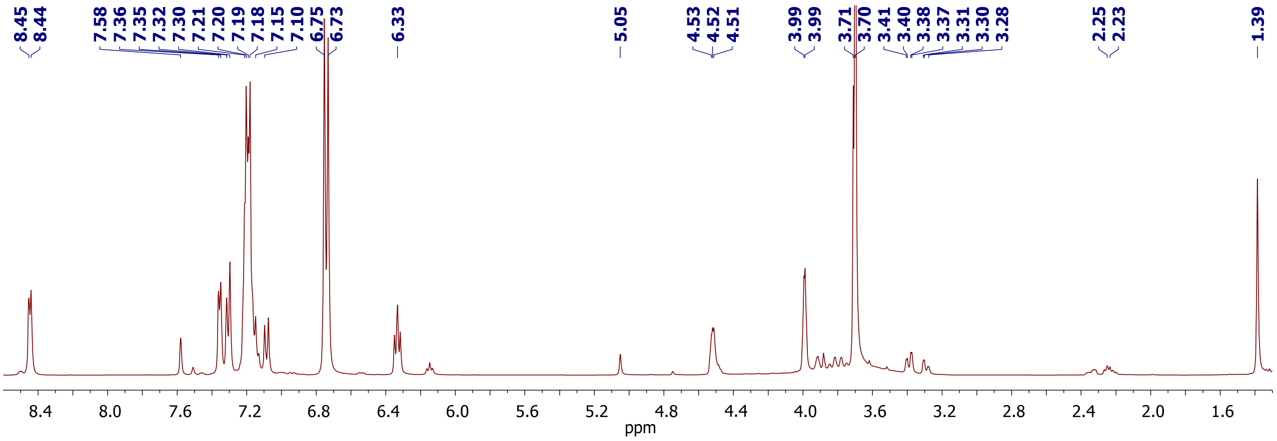

Figure S3. ^{1}H-NMR spectrum of compound **2** (400 MHz, $CDCl_3$).

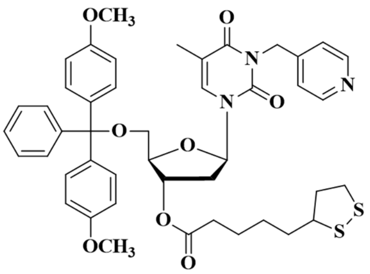

3

Synthesis of 3-(4-pyridylmethyl)-3′-O-lipoyl-5′-O-(4,4′-dimethoxytriphenylmethyl)-thymidine

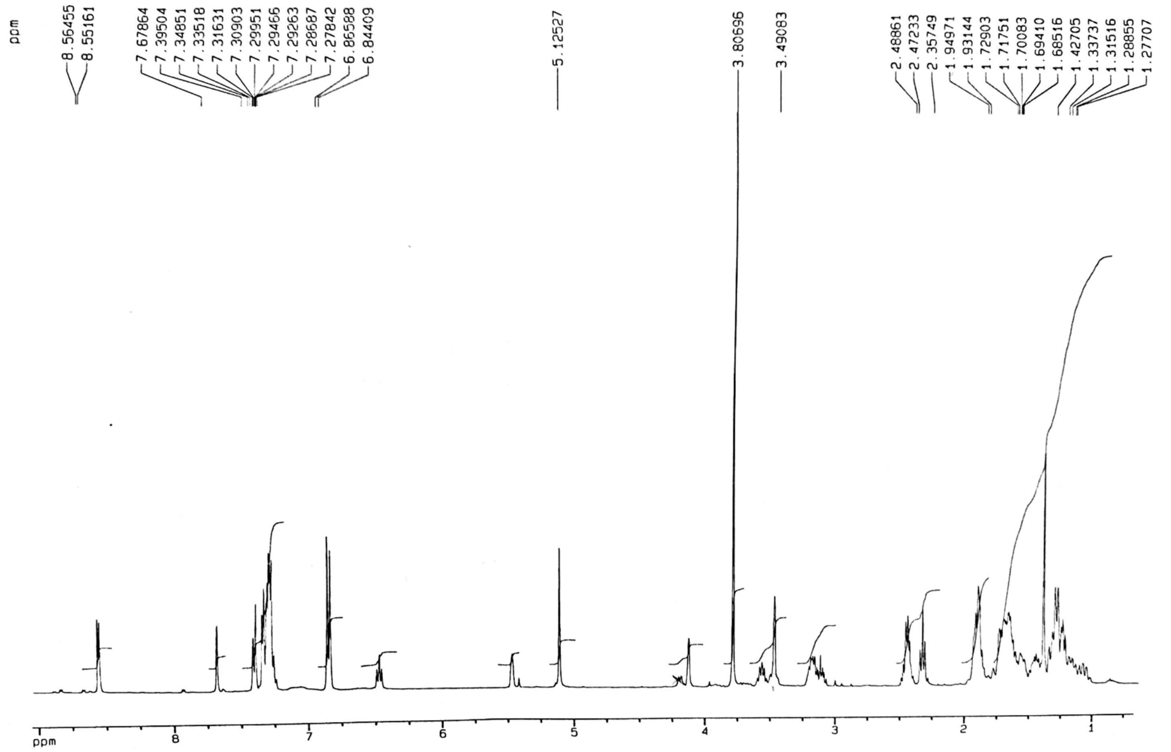

Figure S4. ^{1}H-NMR spectrum of compound **3** (400 MHz, $CDCl_3$).

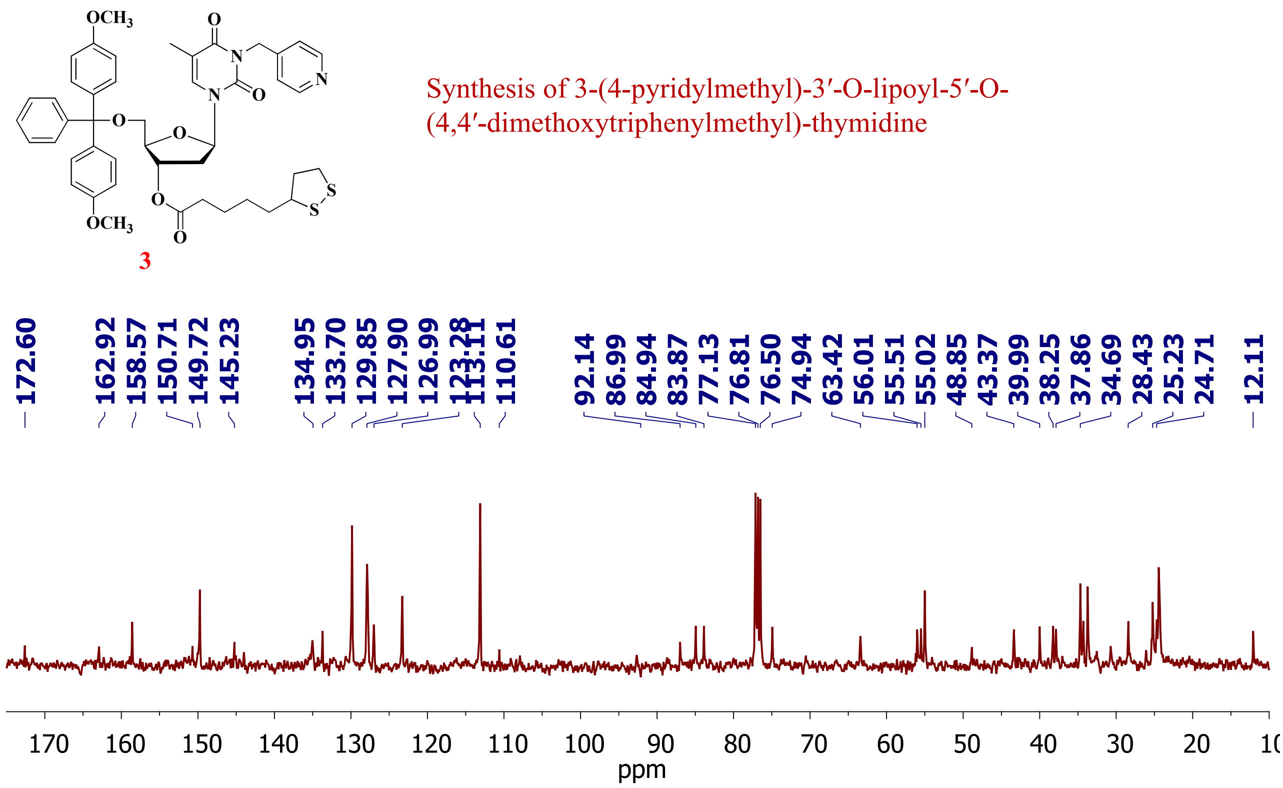

Figure S5. ^{13}C-NMR spectrum of compound **3** (100 MHz, $CDCl_3$).

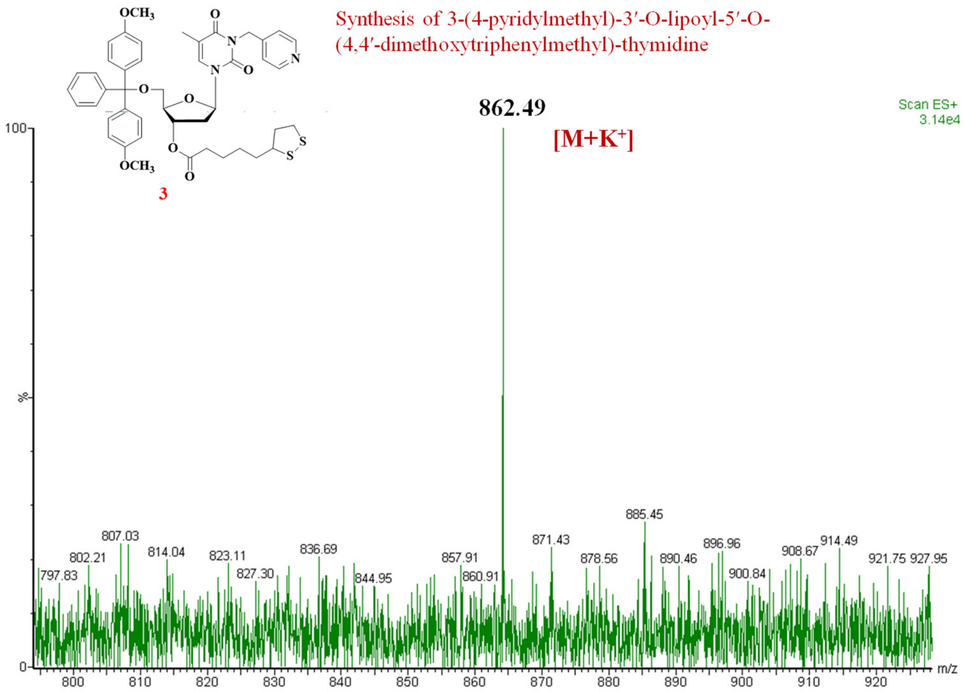

Figure S6. ESI-MS spectrum of compound **3** (positive ions).

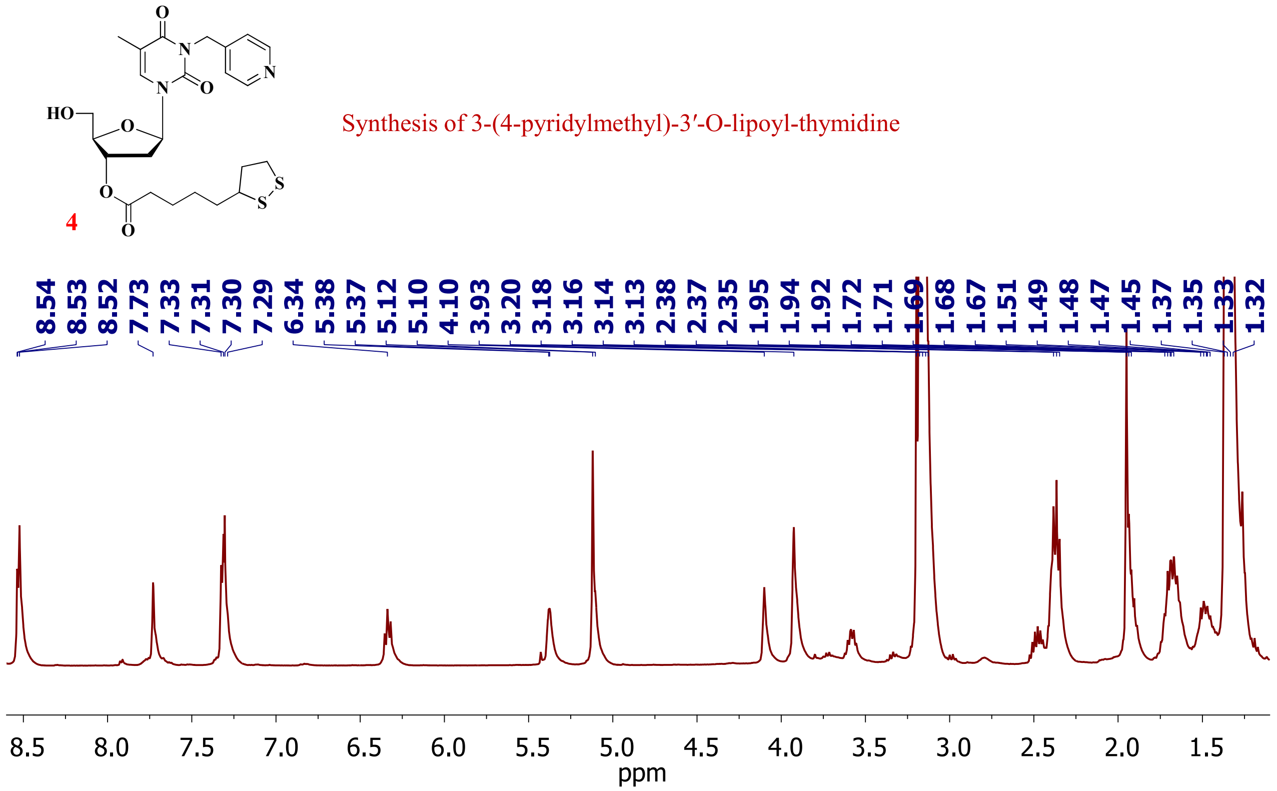

Figure S7. ^{1}H-NMR spectrum of compound **4** (400 MHz, $CDCl_3$).

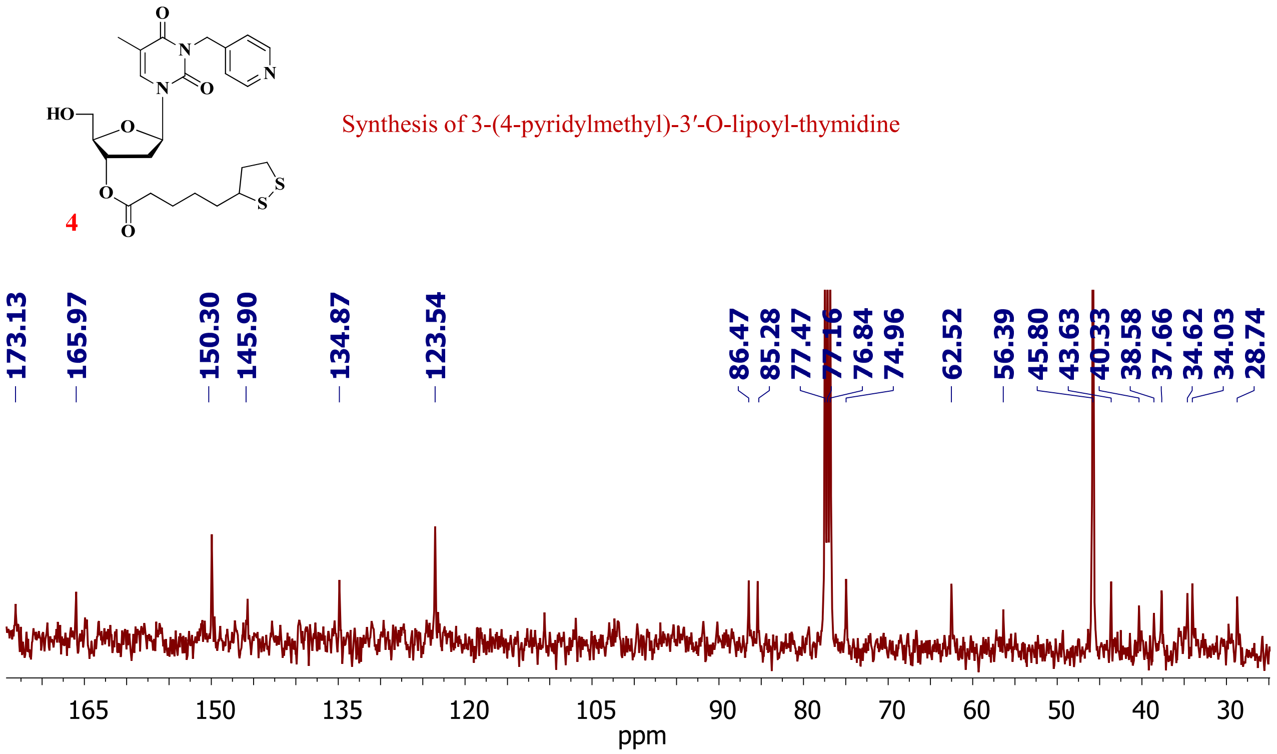

Figure S8. ^{13}C-NMR spectrum of compound **4** (100 MHz, $CDCl_3$).

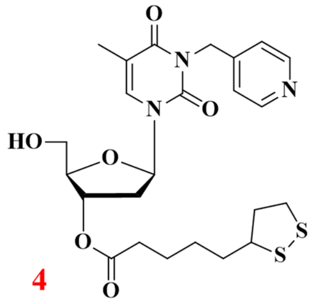

4

Synthesis of 3-(4-pyridylmethyl)-3′-O-lipoyl-thymidine

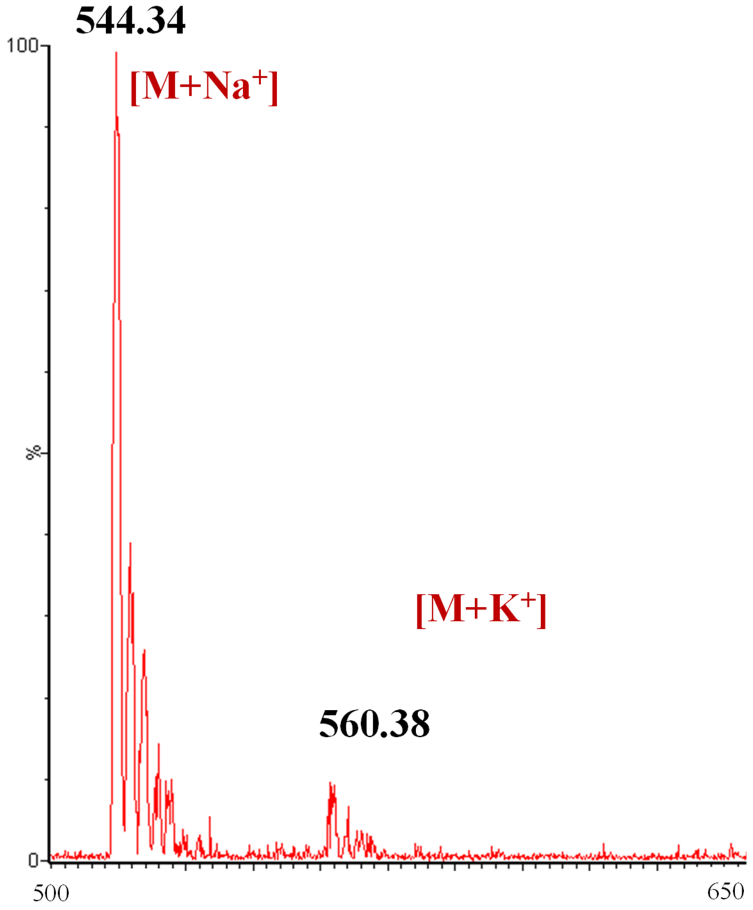

Figure S9. ESI-MS spectrum of compound **4** (positive ions).

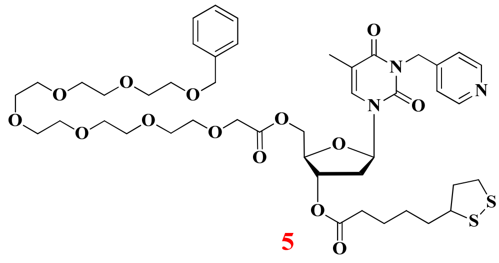

Synthesis of 3-(4-pyridylmethyl)-3′-O-lipoyl-5′-O-(benzyloxy)hexaethylene glycol acetyl-thymidine

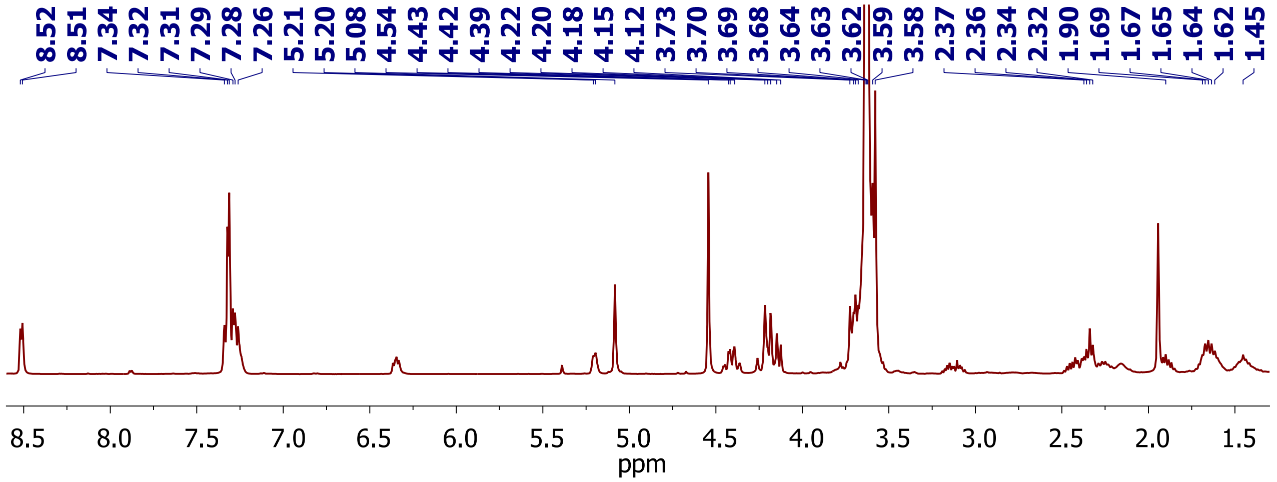

Figure S10. ^{1}H-NMR spectrum of compound **5** (400 MHz, $CDCl_3$).

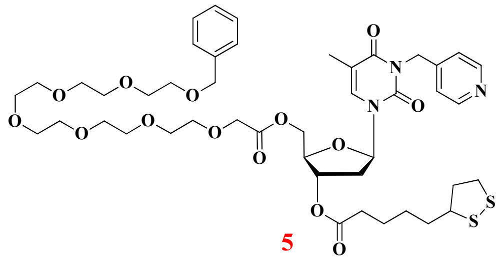

Synthesis of 3-(4-pyridylmethyl)-3′-O-lipoyl-5′-O-(benzyloxy)hexaethylene glycol acetyl-thymidine

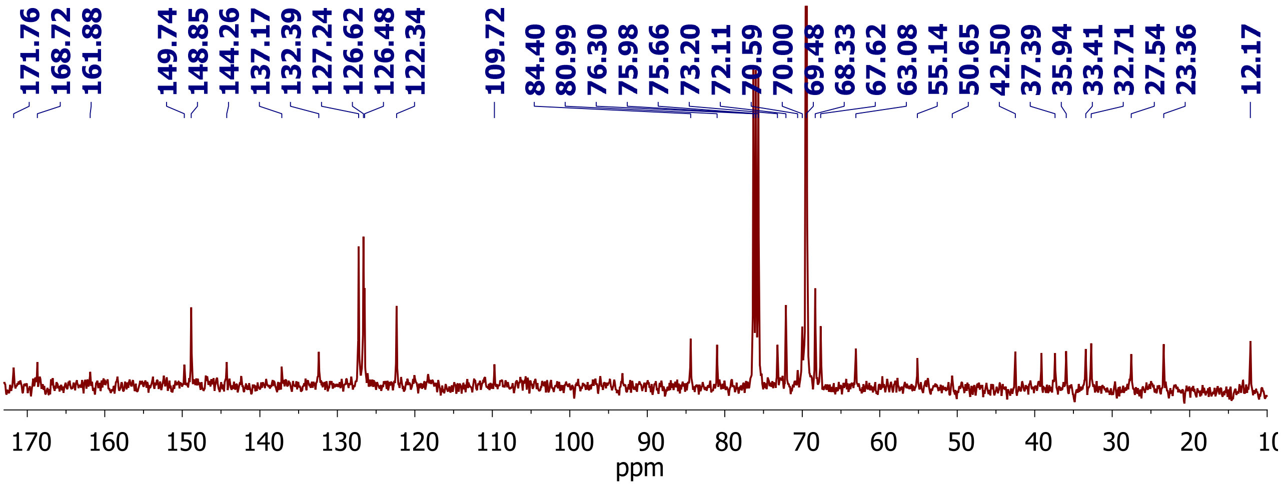

Figure S11. ^{13}C-NMR spectrum of compound **5** (100 MHz, $CDCl_3$).

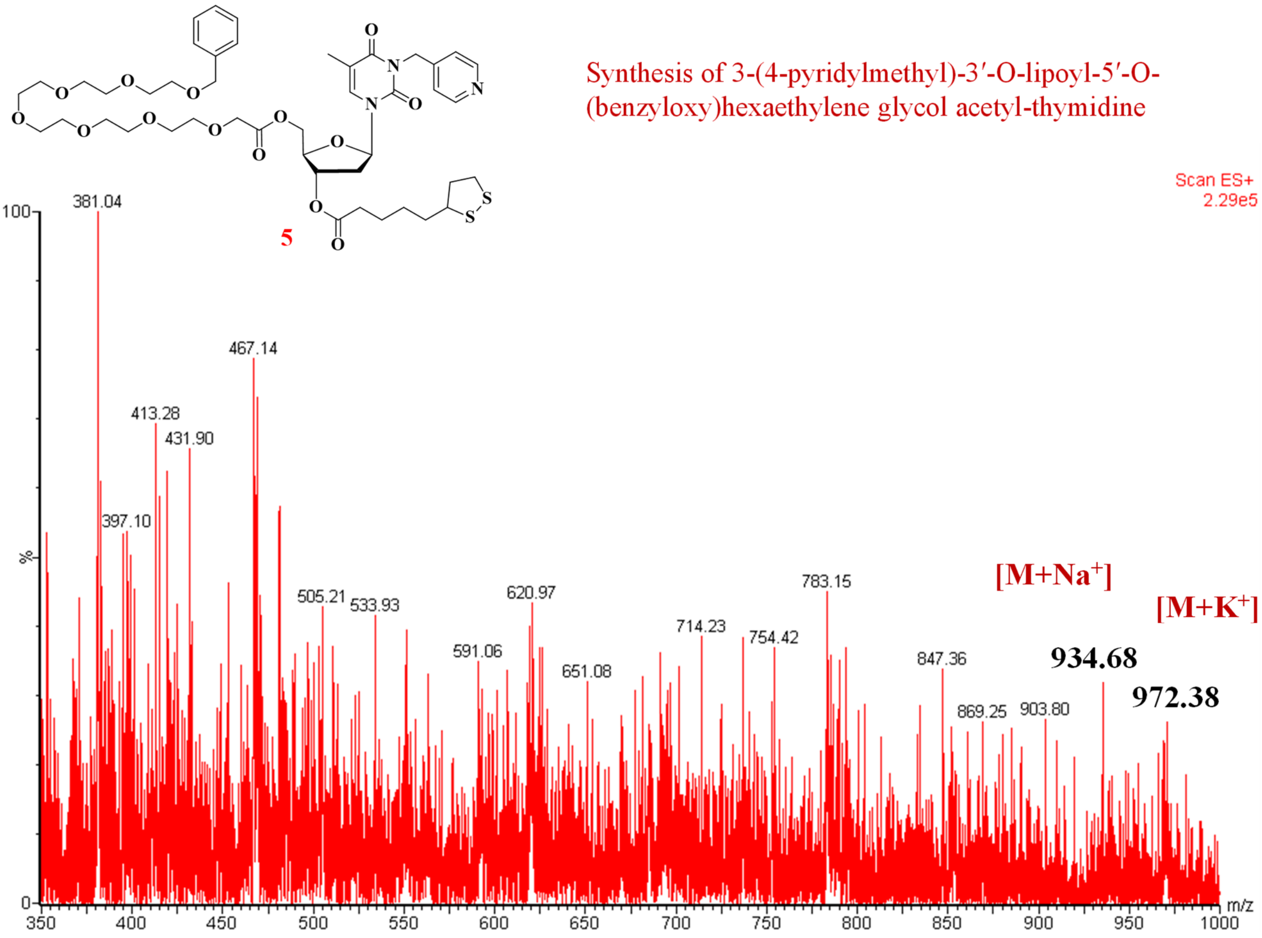

Figure S12. ESI-MS spectrum of compound **5** (positive ions).
